# Ligand-Independent Canonical Wnt Activity in Canine Mammary Tumor Cell Lines Associated with Aberrant LEF1 Expression

**DOI:** 10.1371/journal.pone.0098698

**Published:** 2014-06-02

**Authors:** Ana Gracanin, Elpetra P. M. Timmermans-Sprang, Monique E. van Wolferen, Nagesha A. S. Rao, Juraj Grizelj, Silvijo Vince, Eva Hellmen, Jan A. Mol

**Affiliations:** 1 Department of Clinical Sciences of Companion Animals, Utrecht University, Utrecht, The Netherlands; 2 Department of Molecular Biology, Radboud University, Nijmegen, The Netherlands; 3 Clinic for Obestrics and Reproduction, Faculty of Veterinary Medicine, Zagreb, Croatia; 4 Department of Anatomy, Physiology and Biochemistry, Swedish University of Agricultural Sciences, Uppsala, Sweden; University of Hawaii Cancer Center, United States of America

## Abstract

Pet dogs very frequently develop spontaneous mammary tumors and have been suggested as a good model organism for breast cancer research. In order to obtain an insight into underlying signaling mechanisms during canine mammary tumorigenesis, in this study we assessed the incidence and the mechanism of canonical Wnt activation in a panel of 12 canine mammary tumor cell lines. We show that a subset of canine mammary cell lines exhibit a moderate canonical Wnt activity that is dependent on Wnt ligands, similar to what has been described in human breast cancer cell lines. In addition, three of the tested canine mammary cell lines have a high canonical Wnt activity that is not responsive to inhibitors of Wnt ligand secretion. Tumor cell lines with highly active canonical Wnt signaling often carry mutations in key members of the Wnt signaling cascade. These cell lines, however, carry no mutations in the coding regions of intracellular Wnt pathway components (APC, β-catenin, GSK3β, CK1α and Axin1) and have a functional β-catenin destruction complex. Interestingly, however, the cell lines with high canonical Wnt activity specifically overexpress LEF1 mRNA and the knock-down of LEF1 significantly inhibits TCF-reporter activity. In addition, LEF1 is overexpressed in a subset of canine mammary carcinomas, implicating LEF1 in ligand-independent activation of canonical Wnt signaling in canine mammary tumors. We conclude that canonical Wnt activation may be a frequent event in canine mammary tumors both through Wnt ligand-dependent and novel ligand–independent mechanisms.

## Introduction

The Wnt signaling is one of the key players during normal mammary gland development as well as during mammary tumorigenesis [1]. The canonical, β-catenin-mediated Wnt signaling is activated by secreted Wnt ligands through activation of transmembrane frizzled (Fzd) receptors and LDL-receptor related protein 5 or 6 (Lpr5/6) co-receptors. This triggers Dishevelled-dependent disruption of the β-catenin-destruction complex in the cytoplasm that is composed of multiple proteins including, glycogen synthase kinase 3β (GSK3β), adenomatous polyposis coli (APC), Axin1 (or Axin2) and casein kinase 1 (CK1). Consequently, stabilized β-catenin translocates to the nucleus where it can associate with the T-cell factor (TCF)/Lymphoid enhancer-binding factor (LEF)-family of transcription factors to regulate the expression of specific target genes. The Wnt signal strength can be negatively regulated by secreted factors that competitively bind Wnt ligands (e.g. secreted Fzd related protein (sFRP)) or by factors that disrupt the assembly of Fzd/Lpr5/6 co-receptor complex (e.g. dikkopf-1) [2].

Many studies have documented active Wnt signaling in mammary tissue based on presence of stabilized β-catenin protein and often in combination with aberrant expression of target genes (e.g. Axin2). Based on these criteria, over 50% of human breast tumor tissue samples assessed, showed signs of aberrant canonical Wnt activity (reviewed in [3]). In contrast, reports on the signaling activity in cultured human breast cancer cells are somewhat contradicting. A number of studies have used the presence of active (phosphorylated) or uncomplexed β-catenin in cell lysates as an indicator of active canonical Wnt signaling (Table 1 and references therein). Other studies have applied a more quantitative manner to assess Wnt activity in cultured cells using a TCF-reporter assay. In this assay, the ratio of luciferase signal from reporters containing a promoter with either functional of mutated TCF response elements can be taken as a measure of canonical Wnt activity, with ratios higher than 1.0 indicating an active signaling. Although not always consistent between different studies, a subset of human breast cancer cell lines was found to have moderate TCF-reporter activity (Table 1 and references therein). In addition, a number of studies showed that the effect of canonical Wnt signaling could be attenuated by Wnt inhibitors sFRP1 or Dkk1 [4], [5] or by blocking the receptor Fzd7 [6]. This indicates a ligand-dependent mechanism of canonical Wnt pathway activation in human breast cancer. In concordance, mutations in downstream signaling components (e.g. APC and β-catenin) are rarely found in human primary mammary tumors. Similarly, in cultured cells only one out of 24 screened human breast cancer cell lines (DU4475) had a truncating mutation in APC and none in β-catenin [7]. In contrast, epigenetic silencing of APC and Wnt ligand inhibitors (sFRP1 and Wif1) have often been reported in primary human mammary tumors and in human breast cancer cell lines [8]–[10]. The ligand-dependent nature of canonical Wnt activation in human breast cancer and benign breast lesions is further supported by frequent overexpression of Wnt ligands [3].

**Table 1:** Assessment of canonical Wnt activity in human mammary cell lines.

Cell line	Active or uncomplexed β-catenin	TCF-reporter activity (ratio >1.0)	Reference
AB589	no		[4]
BC3	yes		[4]
BT20		no	[40]
BT474	yes		[41]
BT483		no	[42]
BT549		no	[40], [42]
DU4475		yes	[40]
EVSA-T		no	[40]
HCC1187		yes	[25]
HCC1395		yes	[25]
HCC1937		no	[42]
Hs578T		no	[40]
MCF7	no/yes		[4]/[41], [43]
		no/yes	[40]/[24]
MDA-MB-134	no		[4]
MDA-MB-134VI		no	[40]
MDA-MB-157	yes		[4]
MDA-MB-175	no		[4]
MDA-MB-231	no/yes		[41]/[4], [43]
		no/yes	[25], [40], [44]/[5]
MDA-MB-361	no		[4]
		no	[42]
MDA-MB-415	no		[4]
		no	[42]
MDA-MB-435	no		[4]
MDA-MB-453		no	[40]
	no		[4], [43]
MDA-MB-468	no		[4]
		no	[42]
JIMT-1	yes		[41]
OCUB-F		no	[40]
SkBr3	no		[41]
SK-BR-3		no	[40]
SK-BR-5		no	[40]
SK-BR-7		no	[42]
SUM159		no	[42]
SUM185		no	[42]
T47D	yes		[41], [43]
		no	[40], [44]
UACC893		no	[42]
ZR-75-1	yes		[41]
		no	[42]

Pet dogs have been suggested as a valuable breast cancer model for preclinical research due to the high incidence and spontaneous nature of the tumor development, shared environmental risk factors, strong genetic similarity with humans and shared aspects of mammary tumor biology [11], [12]. Moreover, development of canine mammary tumors is highly dependent on steroid hormone exposure, with progesterone being the main risk factor [13], [14].

The activity of canonical Wnt signaling in canine mammary tumorigenesis has not been quantitatively assessed so far. Previous studies have only addressed the expression of β-catenin protein in spontaneous canine mammary tumors in relation to E-cadherin and/or APC [15]–[17]. However, comparative gene expression profiling of human and canine mammary tumors has implicated a significant similarity in deregulation of multiple cancer-related pathways, including Wnt signaling [18]. In this study we aimed to assess the activation of canonical Wnt signaling in canine mammary tumors using a panel of canine mammary cell lines. We report that subsets of canine mammary tumor cell lines exhibit moderate, ligand-dependent-, and high, ligand-independent-mechanisms of canonical Wnt activation. Moreover, we show that the ligand-independent activation of canonical Wnt signaling is coupled to the overexpression of LEF1.

## Materials and Methods

### Canine mammary cell lines and tissue

Canine mammary tumor cell lines used in this study were established from primary tumors diagnosed as carcinoma (CMT1, CMT-U27, CMT9, P114, CHMp, CNMp and CIPp) or its metastasis (CHMm, CNMm and CIPm), benign mixed tumor (CMT-U229) and osteosarcoma-like tumor (CMT-U335) [19]–[21]. All cell lines were cultured in DMEM/F12 (Invitrogen, Bleiswijk, The Netherlands) supplemented with 10% fetal bovine serum (FBS) (FBS Gold, PAA, Cölbe, Germany). Canine mammary tissue used in this study originates from privately owned dogs that were referred to clinics of Veterinary Faculty in Zagreb, Croatia. Canine mammary surgery was performed as a part of a necessary medical treatment due to the presence of mammary tumor. This was done under the common rules for veterinary surgery for which owners asked for medical treatment of their pets. In contrast to medical intervention in laboratory animals no external permission was necessary other than that the surgery is done by qualified veterinary surgeons. The dog's owners were informed and gave their consent that the collected tissues can be used for research purposes. Histopathology of all tumor and paired normal tissue was evaluated by Prof. E. Hellmen (Table 2). Pictures of cell morphology were captured using an Olympus microscope (Zoeterwoude, The Netherlands) with 10×10 magnification.

**Table 2:** Information about histopathology and RNA quality of canine mammary tumor tissue.

Sample ID	Tumor histopathology	Normal (RIN)	Tumor (RIN)
2	Benign mixed tumor	8.8	8.1
3	Complex adenoma	7.7	8.4
7	Complex adenoma	8.3	8.6
14	Carcinosarcoma (combined osteosarcoma and ductular carcinoma)	7.9	8.3
20	In situ carcinoma	7.6	7.9
25	Atypical sclerosing adenosis and purulent inflammation	7.8	9.2
26	Simple solid carcinoma	8.9	9.0
1	Simple ductal carcinoma	7.1	9.3
5	Simple carcinoma	6.5	8.0
24	Complex carcinoma	8.4	9.8
31	Probable fibrosarcoma/complex carcinoma	8.6	7.1

### TCF-reporter assay

Transfection was performed in FBS-free medium using 3 µl Lipofectamine 2000 (Invitrogen), 800 ng pTOPFLASH (TOP) or pFOPFLASH (FOP) (gift from Dr. Marc van de Wetering, Hubrecht Institute, The Netherlands) and 0.5 ng human β-actin-promoter renilla construct [22] as an internal control. Cells were seeded 48 h before transfection at a density optimal for transfection, according to the manufacturer's protocol in a 24 wells plate (Primaria, BD Biosciences, Breda, The Netherlands). In case of Wnt3a cotransfection, 10 ng mouse pcDNA4-Wnt3a construct (gift from Dr. Wim de Lau, Hubrecht Institute, The Netherlands) was used. Transfection was stopped after 5 h and cells were left to recover for 24 h on DMEM/F12 supplemented with 10% FBS. Cells were then treated with increasing concentrations of IWP-2 (Stemgent, Cambridge, UK) or 5 mM LiCl for 24 h. Control DMSO concentration reflected the DMSO concentration in the 10 µM IWP-2 solution. The firefly and renilla luciferase activities were measured using a Dual-Luciferase Assay System (Promega, Leiden, The Netherlands) in a Centro LB 960 luminometer (Berthold Technologies, Vilvoorde, Belgium). Differences in pTOPFLASH/pFOPFLASH were statistically assessed using unpaired, two tailed Student's t test in Microsoft Office Excel. All transfection experiments were performed using three replicate samples and each experiment was independently repeated 2–4 times.

### siRNA

Canine sequence-specific LEF1 (synonym: TCF1-alpha, Genbank: XM_003434032) and β-catenin (CTNNB1, Genbank: NM_001137652) siRNA was designed on the website http://www.dharmacon.com/designcenter/designcenterpage.aspx (DharmaconRNAi technologies, ThermoScientific, USA). Universal MOCKsiRNA (ON-TARGET plus non-targeting pool species H, M, R) was used as the negative control for siRNA experiments. There was no cross-silencing of non-target genes checked by blasting the siRNA designed sequences against the canine genome database. The sequence of the LEF1 siRNA duplex is as follows: sense GAAGAGGAAGAGAGAGAAAUU and antisense UUUCUCUCUCUUCCUCUUCUU, and for β-catenin sense GAACGAAGGUGUAGCAACAUU and antisense UGUUGCUACACCUUCGUUCUU. Cell transfections were first optimized with siGLO (Dharmacon, Colorado) (data not shown). 80.000 CMT-U27 cells were transfected with 1 µl DharmaFECT Duo as transfection reagent (Dharmacon, Colorado), 50 nM siRNA and 0.5 µg DNA (TOP or FOP) in 24 wells plates (Primaria, BD, The Netherlands). After 24 h and 48 h incubation in DMEM:F12 and 10% FCS cells were harvested for RNA isolation or TCF-reporter assay.

### RNA isolation, cDNA synthesis, sequencing and quantitative RT-PCR

From all cell lines, total RNA was isolated from two different passages. From canine mammary tissue and from the mammary cell lines total RNA was isolated and treated with deoxyribonuclease using the RNeasy mini kit (Qiagen, Venlo, The Netherlands) according to the manufacturer's protocol. Quality of mRNA from tissue samples were assessed using a 2100 Bioanalyzer (Agilent Technologies, Amstelveen, The Netherlands) and RNA integrity number (RIN) of each sample is presented in Table 2. cDNA synthesis was performed using iScript kit (Bio-Rad Laboratories) according to manufacturer's protocol. Specific primer sets were used to amplify gene products for quantitative RT-PCR (Table 3) and sequencing (Table 4). Quantitative RT-PCR was performed using Bio Rad MyIQ detection system (Bio-RAD Laboratories) with SYBR Green Fluorophore. Relative target gene expression was normalized to that of the reference gene RPS19 using a delta Ct method [23], and relative induction of gene expression was statistically assessed using paired, two tailed Student's t test in Microsoft Office Excel. For comparison of relative gene expression between three sets of cell line, REST-MCS beta software was used (http://www.gene-quantification.de/rest-mcs.html). For the sequence reactions we used a standard amplification with Phusion-Hot Start Taq (Finnzymes, Espoo, Finland) according to manufacturer's protocol. DNA sequence reactions were performed using BigDye v3.1 according to the manufacturer's protocol (Applied Biosystems, Foster City, CA). All amplifications were performed on an ABI 3130XL (Applied Biosystems, Foster City, CA) and analyzed in Lasergene (version 9.1 DNASTAR). The obtained sequences were compared with DNA sequences in databases using BLASTn (http://blast.ncbi.nlm.nih.gov/Blast.cgi).

**Table 3:** Information about primers used to assess gene expression.

Gene name	Forward primer	Reverse primer	Annealing T (°C)
SFRP1	AGCGAGTTTGCATTGAGGAT	TCTTGATGGGTCCCAACTTC	60
APC	AGTCCCAAGCAACAGAAGC	GCAGTTGAACCCTGAGCA	63
β-catenin (CTNNB1)	ATGGGTAGGGCAAATCAGTAAGAGGT	AAGCATCGTATCACAGCAGGTTAC	64
E-cadherin (CDH1)	CAGGAAGCTCTCCACCAGAG	CTGGGAAATGTGAGCACCTC	58
LEF1	AGACATCCTCCAGCTCCTGA	GATGGATAGGGTTGCCTGAA	60
TCF1 (TCF7)	CTACTCCGCCTTCAATCTGC	AGAGAGTTGTGGGACGCTGT	60
TCF3 (TCF7L1)	CCTGGAGCTGTTGGACAAAT	AAACCAGGCTGGACATTGAG	60
TCF4 (TCF7L2)	CGAGTGCACGTTGAAAGAAA	ATGTGAAGCTGTCGCTCCTT	60
WNT1	CTGGCACGTTGACTCAGAGA	AAGAGCTGCATAGCCACCAC	63
WNT2	GACAGGGATCACAGCCTCTT	TGGTGATGGCAAACACAACT	63
WNT3	ATGAACAAGCACAACAACGAG	TTGAGGAAGTCGCCGATAG	61.5
WNT4	CGAGGAGTGCCAGTACCAGT	AGAGATGGCGTACACGAAGG	61,6
WNT5B	CCCTGTACAGAGACCCGAGA	ACAACTGGCACAGCTTCCTC	61.5
WNT7A	GCCTCGACGAGTGTCAGTTT	GATGATGGCGTAGGTGAAGG	60
RPS19	CCTTCCTCAAAAAGTCTGGG	GTTCTCATCGTAGGGAGCAAG	61
AXIN2	GGACAAATGCGTGGATACCT	TGCTTGGAGACAATGCTGTT	60

**Table 4:** Information about primers used to sequence gene coding regions.

gene name	Forward primer	Location	Reverse primer	Location
β-catenin	AAGCACACCATACAACGG	F4	CCTAAACCACTCCCACCCT	R2494
(CTNNB1)	GGCTGCTATGTTCCCTGAGA	F327	CCACCTGGTCCTCATCATTA	R541
	GGGACCTTGCACAATCTTTCTC	F688		
	AATGCAGGCTTTAGGGCTTCA	F1128		
	CCTGCCATCTGTGCTCTTCGTC	F1429		
	TCACAACCGAATCGTAATCAGA	F1794		
APC	AGAGGCAGACTCAGCACCAT	F3728	GGGGCTTATAATGCCACTCA	R4311
	GGCATTATAAGCCCCAGTGA	F4297	ACAGGGGGAGGTAATTTTGG	R4865
	AAAGAGCCCGAAAAGCCTAC	F4702	ACACGGAAAGGCTTGTGACT	R5279
	GCCCAAAGGAAAAAGTCACA	F5247	CGATTTACGGGGTGTTTTGT	R5810
	CCAGGGAAAAGGCTGAATTA	F5675	ACTCCTGCAACAGGTCGTCT	R6178
	AGACGACCTGTTGCAGGAGT	F6159	GGGCTGTTTCATTTGGCTTA	R6702
	AGCAAACATGCCTTCGATCT	F6708	CCTTTGGAGGCAGACTCACT	R7253
	ACGTCTCCAGGCAGACAGAT	F7153	GAATGGGAGCGTGCAATATC	R7652
	CACGCTCCCATTCTGAAAGT	F7640	CCGTTACCCACACTTGGTTT	R8180
	GAGATCCCCAACAGGAAACA	F8070	CACACGGATGTCACGAGGTA	R8585
GSK3B	GAGGGTGATTCGGGAAGAG	F1005	TAGGCTAAACTTCGGAACAG	R1542
	CGGAAACAGTATACAGAGTTGC	F1437	AAGTAACTGGTGGTTCTTCCTG	R2338
	TTCCCTCAAATTAAGCAC	F1907		
CK1	GAGCGGCGGCGATCAGGTTCC	F217	ATACCCATTAGGAAGTTATCTGGT	R835
(CSNK1A1)	TACACAGAGACATTAAACCAGATA	F796	ATCTGCTCTGCTTCTTCTGTTC	R1446
	ATAAAAATCTCACTGGCACTG	F1016		
AXIN1			CTTCGTTCAGAGTGGGCAGGTAGC	R730
	GTTTGACCAGGCACAGACGGAGAT	F543	CGGTAAGTGCGAGGAATGTGAGGT	R1127
	ACCGACAGCAGCGTGGAT	F1006	ACTGTGGTGACTGTGGTGGTG	R1626
	CCGGCCATCGTTCCCCTGACAAT	F1466	GCGGTGCCTGCTGATCTCCTTCTC	R1941
	TCGGAGGACACAGACAAGAAC	F1867	GAACCTCCTCGAACACCACTC	R2536
	GCGGAGTGGTGTTCGAGGAGGTTC	F2513		
E-cadherin			TCTGGTTTATGAAGTTGTAGAGGC	R399
(CDH1)	CAGCCTATGTTTCTGATGAC	F312	ACAGATCCTTGGAAGACTGC	R921
	AGTCTTCCAAGGATCTGTCAC	F904	CAGTCACGTACAAGACATACTG	R1475
	TTTGTTGTCACCACAGACCC	F1376	CAATGATGTTGATGACATGAGG	R1955
	ACCTCGAAATATGGACTTCTG	F1897	CAACTGGCTCAAGTCAAAGTC	R2428
	CTCGCTCTACTAATCCTGATTCTG	F2264	CCATTCATTCAGGTAGTCATAGTC	R2707
			TTCTCCAGAAATTCCTCTCAG	R2861
LEF1	GAGCGGAGATTGCAGAGC	F615	CGTTGGGAATGAGTTTCGTT	R1875

### Protein extraction and Western blot

For whole cell lysis cells were washed with cold HANK's balanced salt solution and scraped in cold RIPA buffer (6.5 mM Na2HPO4, 1.5 mM KH2PO4, 137 mM NaCl, and 2.7 mM KCl (pH 7.4); 1% sodium dodecyl sulfate (SDS) (vol/vol), 1% Igepal (vol/vol), 0.5% Na-deoxycholate (wt/vol), 1 mM phenylmethylsulfonylfluoride,1 mM Na-orthovanadate and 1 µg/ml aprotinin). After 20 min incubation on ice, samples were centrifuged for 15 min at 16,000 g at 4°C. Protein concentration was determined using Bio-Rad Dc Protein Assay (Bio-Rad Laboratories). Fifty microgram protein of total cell lysates was subjected to SDS-PAGE and analyzed by Western blot. For extraction of cytoplasmic and nuclear protein fractions NE-PER Reagent kit (Thermo Scientific, Breda, The Netherlands) was used according to the manufacturer's protocol. Ten µg protein was subsequently subjected to western blot analysis. Primary antibodies used in this study were directed against APC (AB-1) Mouse (FE9) (OP44 1∶1000), (Calbiochem, Merck, Amsterdam, The Netherlands), β-catenin (Ab6302 1∶4000)(Abcam, Cambridge, UK), human E-cadherin (610181, 1∶2000 BD Biosciences, Breda, The Netherlands), GAPDH (ab9485 1∶2000) (Abcam) and β-actin pan Ab-5 (MS-1295-P1 1∶2000)(Thermo Scientific) as a reference protein. And as secondary antibody goat anti-mouse HRP-conjugated (HAF007, R&D Systems, Abingdon, UK) was used. HRP was visualized using Advance TM_Enhancedchemiluminescence (ECL, Amersham, GE Healthcare, Eindhoven, The Netherlands) and analyzed using GelDoc2000 (BioRad).

## Results and Discussion

### Canonical Wnt signaling is aberrantly active in a subset of canine mammary tumor cell lines

**Figure 1:**
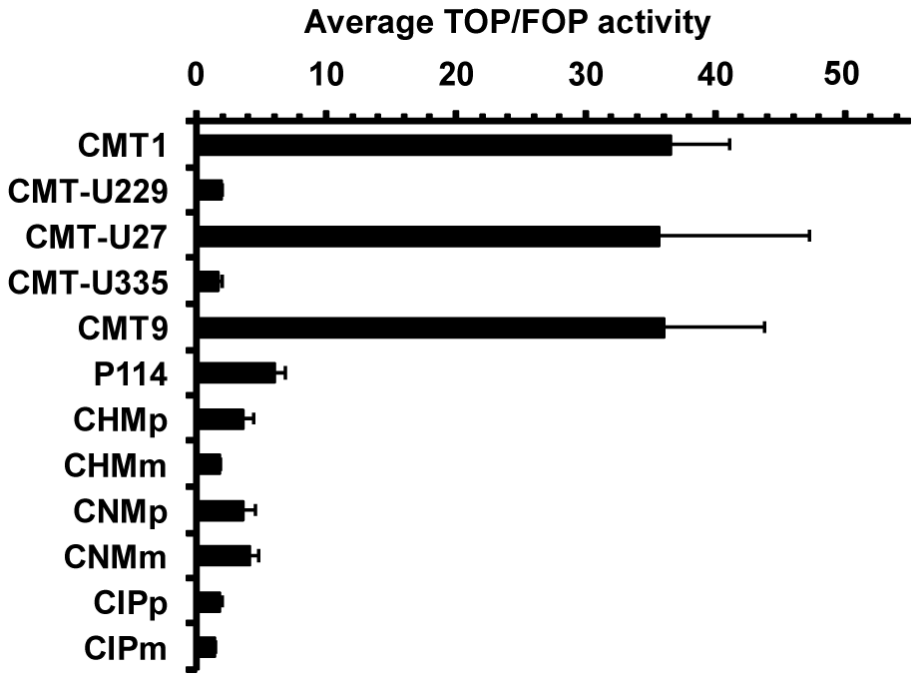
TCF-reporter activity in canine mammary tumor cell lines. Average TOP/FOP ratio (+/− s.e.m.) in canine mammary cell lines from 3–4 independent experiments.

To quantitatively assess canonical Wnt activity we tested a panel of 12 canine mammary cell lines using a TCF-reporter assay (Fig. 1). Three cell lines (CMT1, CMT-U27 and CMT9) showed high TCF-reporter activity. Four cell lines (P114, CHMp, CNMp and CNMm) showed moderate reporter activity comparable to previously reported activity in human mammary cell lines [24], [25]. The remaining five cell lines (CMT-U229, CMT-U335, CHMm, CIPp and CIPm) with the TOP/FOP ratio around 1, lacked canonical Wnt activity.

### Ligand-dependent and -independent mechanism of canonical Wnt activation in canine mammary cell lines

**Figure 2:**
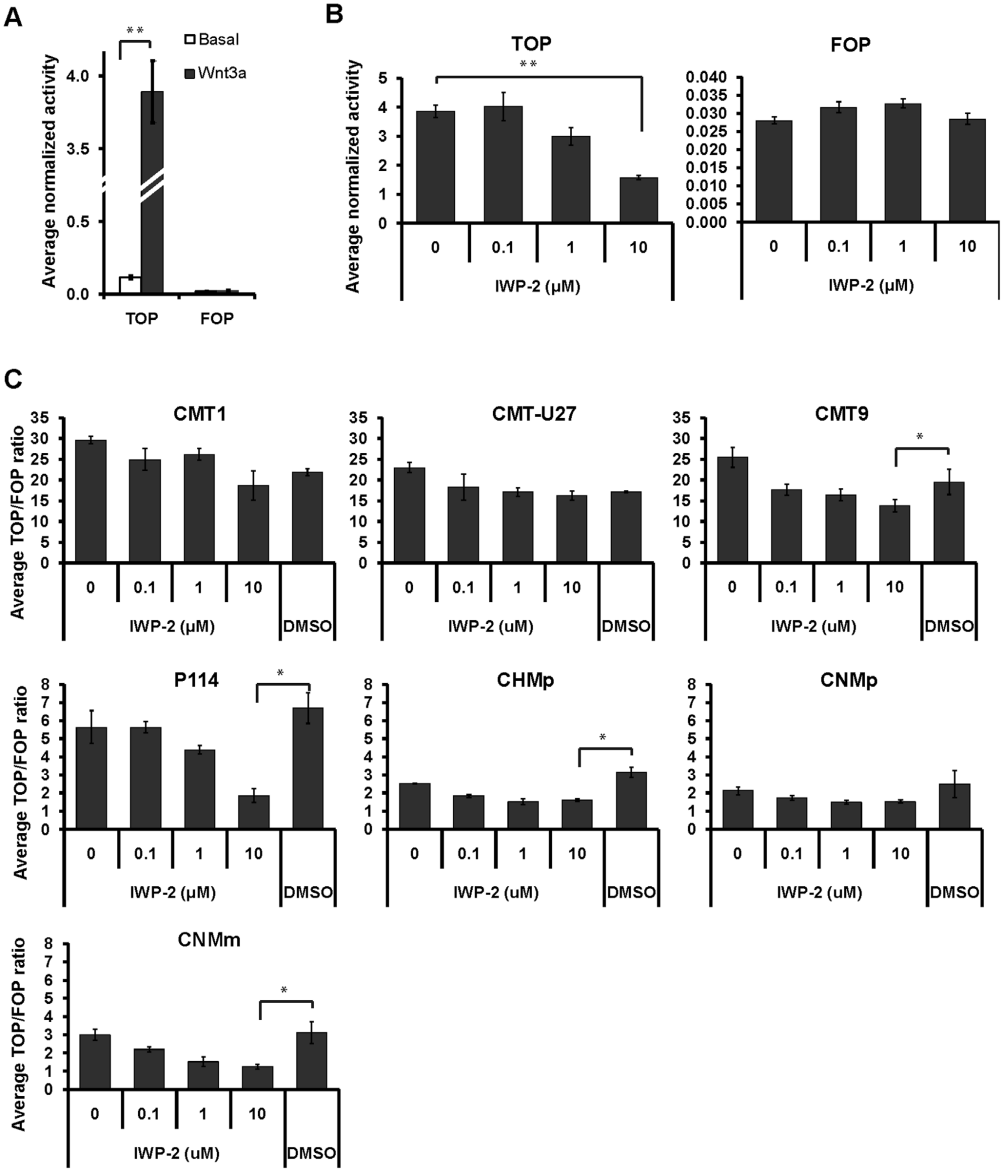
Inhibition of canonical Wnt activity using porcupine inhibitor IWP-2. (A) Effect of transient Wnt3a co-transfection on TOP and FOP activities in P114 cells. (B) Effect of IWP-2 treatment on TOP and FOP activities in P114 cells co-transfected with Wnt-3a. (C) Effect of IWP-2 treatment on basal TOP/FOP ratio in CMT1, CMT-U27, CMT9, P114, CHMp, CNMp and CNMm cell lines. TOP/FOP ratio after treatment with 10 µM IWP-2 was tested against the control DMSO treatment. * indicates p<0.05 and **p<0.01.

**Figure 3:**
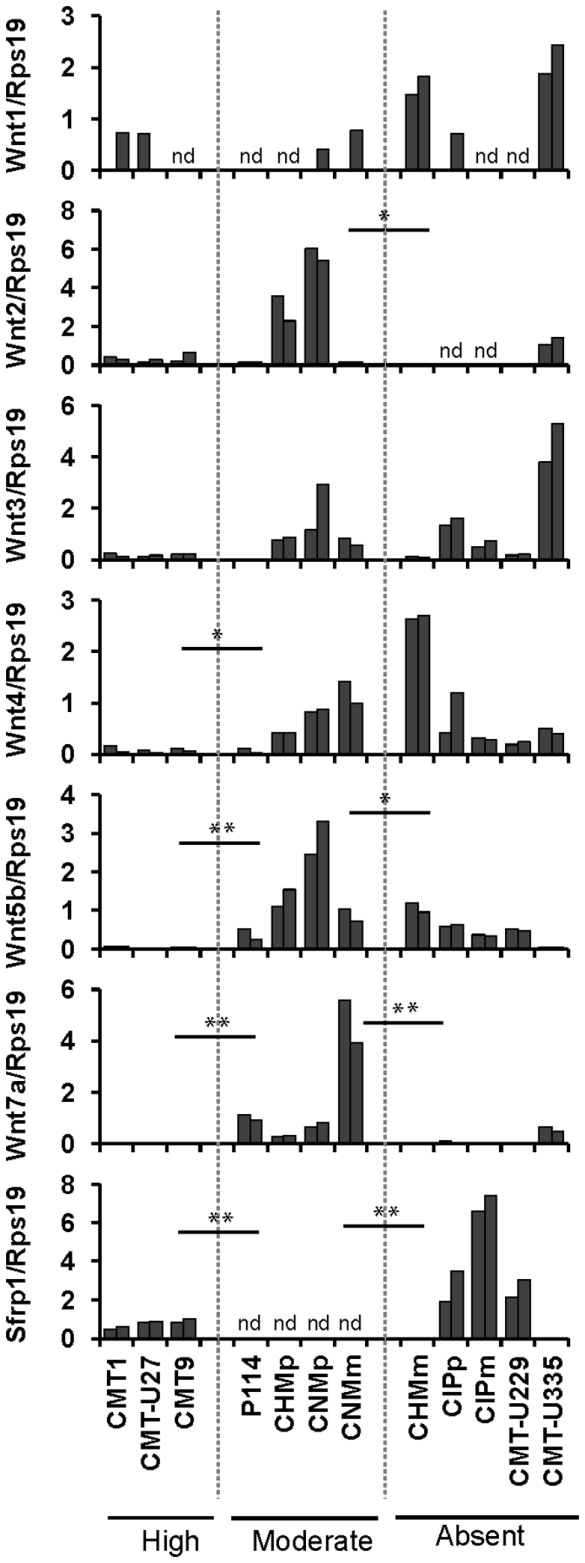
Expression of multiple Wnt ligand- and inhibitor Sfrp1-mRNA. mRNA expression of Wnt1, Wnt2, Wnt3, Wnt4, Wnt5b, Wnt7a and Sfrp1 in two different passages of cell lines. Target gene expression was normalized to that of a reference gene Rps19. Cell lines were divided in three groups (from left to right): cell lines with high, moderate or absent canonical Wnt activity. * indicates p<0.05, **p<0.01 and nd stands for non-detectable.

IWP-2 is a small molecule inhibitor that impairs Wnt ligand palmitoylation and secretion [26] and could, therefore, be used to discriminate between ligand-dependent and -independent mechanisms of canonical Wnt activation in cells. To assess the activity of IWP-2 in canine cells, P114 cell line was transiently co-transfected with canonical Wnt ligand Wnt3a (Fig. 2A) and treated with increasing concentration of IWP-2. IWP-2 effectively inhibited Wnt-3a-dependent TOP-flash activity, but not the FOP-flash activity (Fig. 2B), confirming its specificity as canonical Wnt inhibitor in canine cells. The effect of IWP-2 treatment on the basal canonical Wnt activity was subsequently evaluated in all cell lines with active Wnt signaling. In cell lines with moderate basal canonical Wnt activity (i.e. P114, CHMp, CNMp and CNMm), IWP-2 was able to efficiently inhibit the TCF-reporter activity. Treatment with 10 µM IWP-2 resulted in TOP/FOP ratios around 1, suggesting a full ligand-dependency in the cell lines with moderately activate Wnt signaling (Fig. 2C). Moreover, we have assessed the expression of several Wnt ligands previously reported as activators of canonical signaling and/or being expressed in mammary tissue and cell lines [27], [28]. Ligand-dependent activation of the pathway in these cell lines is further supported by the high expression of multiple Wnt ligands (especially Wnt5b and Wnt7a) and undetectable levels of the inhibitor sFRP1 (Fig. 3). IWP-2 treatment in CMT1, CMT-U27 and CMT9 cells, however, had no or only a minor effect on the TCF-reporter activity (Fig. 2C). These three cell lines are therefore expected to have a ligand-independent component for canonical Wnt activation. Recently, it has been reported that conditioned medium of tumor-associated macrophages or co-culture with macrophages mediate a switch from canonical to non-canonical Wnt signaling in multiple canine mammary cell lines, including P114 and CMT-U27 [29]. Inhibition of canonical Wnt signaling (demonstrated by downregulation of cytoplasmic and nuclear β-catenin protein levels) was associated with exposure of cells to increased levels of non-canonical Wnt ligands and canonical Wnt inhibitor Dkk-1. Our data supports the responsiveness of P114 cell line to Wnt ligands and inhibitors. However, the insensitivity of basal TCF-reporter activity in CMT-U27 cells to treatment with IWP-2 suggests that the reported inhibition of canonical Wnt signaling [29] is not mediated by altered Wnt ligand or inhibitor expression, but most probably is caused by other mechanisms.

### High canonical Wnt activity is not associated with a lack of functional E-cadherin

**Figure 4:**
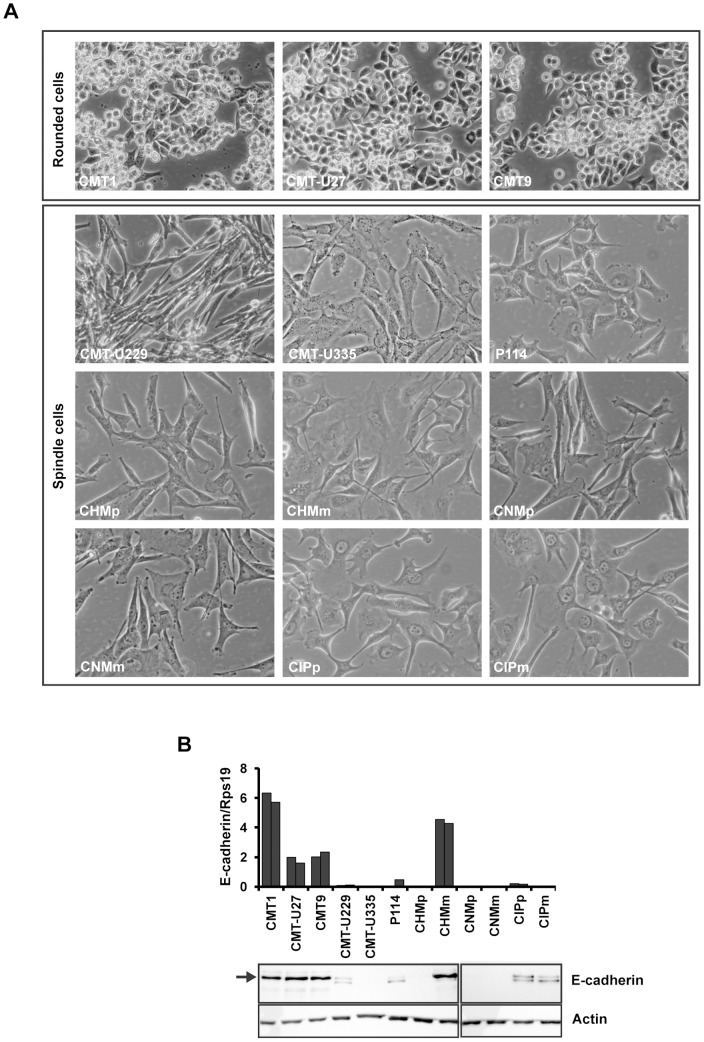
Cell line morphology and E-cadherin expression. (A) Canine mammary cell lines grouped based on their morphology as (partially) rounded cells or spindle cells. (B) Expression of E-cadherin at mRNA (top) and protein (bottom) level. Rps19 and actin expression served as reference mRNA and protein, respectively. Arrow indicates the position of full-length mature E-cadherin protein. Additional E-cadherin protein band present in some of the cell lines represents the unprocessed form of the protein.

The majority of the canine mammary cell lines used in this study had spindle-cell-like morphology, except for CMT1, CMT-U27 and CMT9, which grow as attached cells but also as partially rounded cells (Fig. 4A). A partially rounded morphology has been associated with E-cadherin mutations in human breast cancer cell lines [30]. As the loss of E-cadherin protein can stimulate canonical Wnt activity [31], we analyzed its coding sequence and mRNA and protein levels in all 12 canine mammary cell lines. Sequencing analysis of the whole CDH1 coding region revealed no mutations in any of the cell lines (Table S1). Moreover, CMT1, CMT-U27 and CMT9 highly expressed mRNA and mature protein of E-cadherin (Fig. 4B), suggesting a different mechanism of canonical Wnt activation in these cell lines.

### High canonical Wnt activity is not associated with defects in β-catenin destruction complex

**Figure 5:**
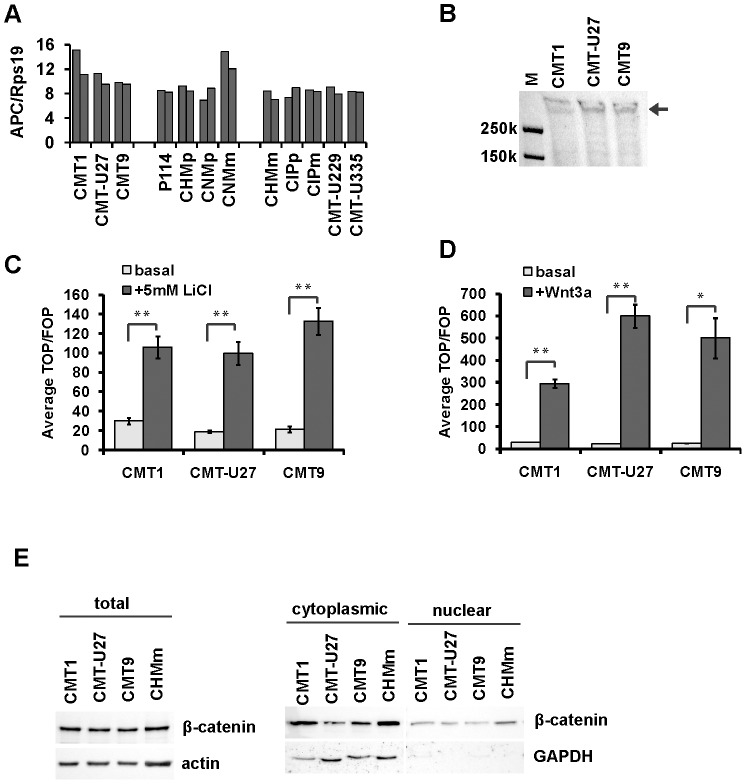
Assessment of defects at the level of β-catenin destruction complex. (A) mRNA expression of APC in two different passages of canine mammary cell lines normalized to the expression of Rps19. (B) APC protein expression in CMT1, CMT-U27 and CMT9. M indicates loading marker with reference molecular weight bands. (C) Effect of treatment with 5 mM LiCl or co-transfection with Wnt-3a (D) on TCF-reporter activity in CMT1, CMT-U27 and CMT9. * indicates p<0.05 and **p<0.01. (E) β-catenin protein expression in total cell lysates (total) and the cytoplasmic and nuclear fractions.

In multiple tumors, elevated canonical Wnt activity has been shown to result from mutations in components of β-catenin destruction complex [32]. Mutational analysis of coding sequences of APC, β-catenin, GSK3β, CK1α and Axin1 in canine mammary cell lines revealed, however, no mutations that were restricted to cell lines with the active canonical Wnt signaling (Table S1). As APC is also known to be epigenetically silenced or proteolytically cleaved in tumors [33], [34], its mRNA and protein expression were additionally assessed. All 12 canine mammary cell lines expressed comparable levels of APC mRNA (Fig. 5A). Analysis of protein expression in CMT1, CMT-U27 and CMT9 revealed that APC was expressed as a full-length protein (Fig. 5B). We next asked whether high canonical Wnt activity in these cells is a consequence of a defect at the level of β-catenin destruction complex. Canonical Wnt signaling in cells in which β-catenin destruction complex function is fully impaired is expected to be insensitive to further stimulation of the pathway by Wnt ligands or to treatment with GSK3β inhibitors [35]. CMT1, CMT-U27 and CMT9 cells, however, responded potently to GSK3β inhibitor, LiCl (Fig. 5C) as well as to Wnt3a transfection (Fig. 5D). To determine whether the high Wnt activity is associated with increased stabilization of β-catenin protein we assessed total, cytoplasmic and nuclear levels of β-catenin by western blot. GAPDH was used as a marker of cytoplasmic proteins to assess the purity of extracts from different cell fractions. When compared to a cell line lacking canonical Wnt activity (CHMm), CMT1, CMT-U27 and CMT9 cells did not show evidence of marked cytoplasmic or nuclear β-catenin protein stabilization (Fig. 5E), implying no major defect in the β-catenin destruction complex function.

### Ectopic LEF1 expression contributes to the high canonical Wnt activity in CMT1, CMT-U27 and CMT9

**Figure 6:**
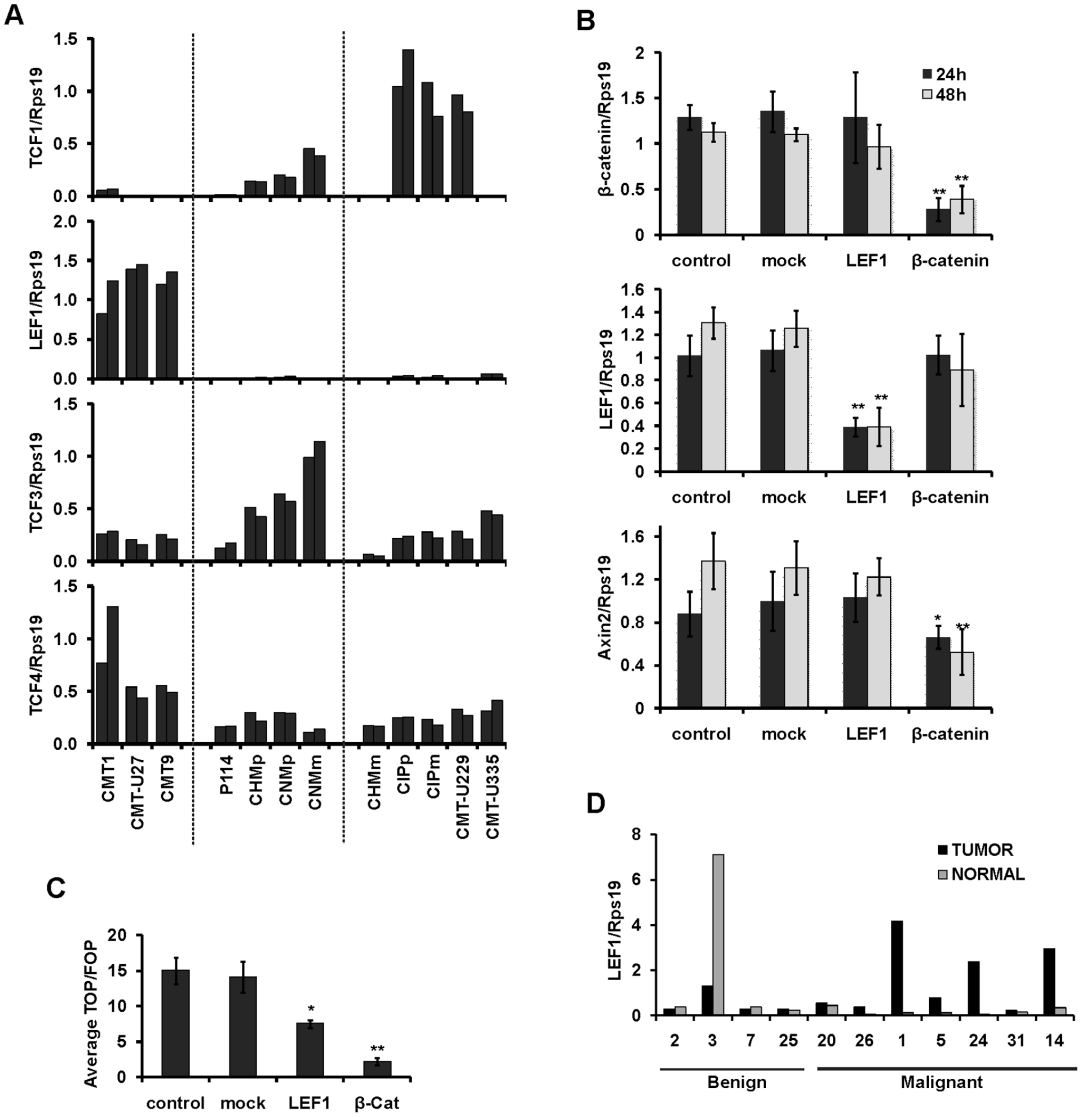
Association between high canonical Wnt activity and LEF1 expression. (A) mRNA expression of TCF1, LEF1, TCF3 and TCF4 in two different passages of canine mammary cell lines normalized to the expression of Rps19. (B) β-catenin, LEF1 and Axin2 mRNA expression in CMT-U27 cells that were either non-transfected (control) or transfected with mock control (mock), LEF1 siRNA (LEF1) or β-catenin siRNA (β-catenin). mRNA expression was analyzed 24 h and 48 h post-transfection. (C) Average TOP/FOP ratio in CMT-U27 cells as described in (B). * indicates p<0.05 and **p<0.01 compared to the mock control. (D) Rps19 normalized mRNA expression of LEF1 in a panel of canine mammary tumors (tumor) and normal mammary tissue (normal) from the same dog.

Upon stabilization, β-catenin translocates to the nucleus and interacts with members of TCF/LEF family of transcription factors (TCF1, LEF1, TCF2 and TCF4). Interestingly, although all TCF/LEF family members were expressed in canine mammary cell lines, LEF1 showed high mRNA expression specifically in CMT1, CMT-U27 and CMT9 (Fig. 6A). Different LEF1 isoforms have been described, resulting from alternative splicing of LEF1 transcript [36]. Sequencing of LEF1 coding region in CMT1, CMT-U27, CMT9 and CIPp showed that the first three cell lines express LEF1 transcript lacking exon 6. Lack of exon 6 in Xenopus LEF1 has been shown to lower its transcriptional potential on TOPFlash reporter in HEK293 cells [37]. Lack of exon 6 in LEF1 in CMT1, CMT-U27 and CMT9 can therefore not be attributing to the high canonical Wnt activity in these cell lines. As LEF1 is known to be a direct target gene of canonical Wnt signaling [38] we asked whether high expression of LEF1 in these three cell lines could be a cause or a consequence of high canonical Wnt activity. For this purpose β-catenin and LEF1 knock down was performed in CMT-U27 cells. Knock-down of β-catenin resulted in a potent inhibition of TCF-reporter activity (Fig. 6C) and down regulation of Axin2 target gene (Fig. 6B), but it had no effect on the expression of LEF1 (Fig. 6B). Knock-down of LEF1 did not affect β-catenin expression (Fig. 6B) but was able to significantly inhibit TCF-reporter activity (Fig. 6C). Altogether, this suggests that the ectopic expression of LEF1 in CMT1, CMT-U27 and CMT9 is not a consequence but rather a contributing factor to the high canonical Wnt activity in these cell lines. LEF1 was recently shown to affect the viability, invasion and migration of breast cancer cells [39]. The correlation between LEF1 overexpression and high canonical Wnt activity in canine mammary tumor cell lines prompted us, therefore, to assess LEF1 expression in spontaneous canine mammary tumors. To assess the tumor-specific overexpression of LEF1, each canine mammary tumor was compared to the corresponding normal tissue from the same patient. Interestingly, five out of seven malignant tumor samples showed overexpression of LEF1 (Fig. 6D). However, the sample size will need to be increased in order to test the statistical significance of these results. A challenging task remains to assess whether LEF1 overexpression in canine mammary tumors is associated with high canonical Wnt activity. Considering that canine mammary tumor cell lines with high LEF1 expression do not seem to show marked overstabilization of β-catenin protein (Fig. 5E), an alternative marker for canonical Wnt activity in tissue samples is needed. In this regard, Axin2 mRNA levels were suggested to correlate with mutations in the Wnt signaling pathway in a panel of human cancer cell lines [32]. However, in canine mammary cell lines, basal Axin2 mRNA levels do not correlate with the canonical Wnt activity (Fig. S1), implicating that Axin2 expression is also not a reliable canonical Wnt activity marker in canine mammary tumors. On a further note, LEF1 knock-down was not able to fully inhibit TCF-reporter activity in canine mammary cell lines. This may be a consequence of insufficient knock-down of LEF1 mRNA but it may also argue for involvement of additional canonical Wnt activating factors. To test whether other TCFs may be compensating for the knock-down of LEF1, the levels of all TCF-family members were assessed 24 h after knock-down of LEF1 (Fig. S2). Expression of neither of TCFs showed, however, signs of compensation. Lastly, the use of transient transfection system prevented us from investigating a relationship between LEF1 overexpression and the cellular morphology. For this purpose stable transfection of inducible LEF1 knock-down system should be employed.

## Conclusions

Altogether, this study provides evidence for moderate, ligand-dependent canonical Wnt activation in canine mammary tumors that is comparable to human breast cancer. In addition, we report a novel ligand-independent mechanism involving LEF1 overexpression, which results in high canonical Wnt activity. Our further studies aim to explore this ligand-independent mechanism extensively and to identify the underlying gene mutations.

## Supporting Information

Figure S1 **Axin2 mRNA expression.** Rps19 normalized Axin2 mRNA expression in two different passages of canine mammary cell lines. Cell lines were divided in three groups (from left to right): cell lines with high, moderate or absent canonical Wnt activity.
(TIF)

Figure S2 **Expression of TCF-family members upon LEF1 knock-down.** Relative Rps19 normalized mRNA expression of LEF1, TCF1, TCF3 and TCF4 24 h after LEF1 knock-down in CMT-U27 cells. Average expression of control conditions for each target gene is set to 100.
(TIF)

Table S1 **Sequencing results of target gene coding regions in canine mammary tumor cell lines.**
(DOCX)
