# Supplementary figures and images for: Ligand-Independent Canonical Wnt Activity in Canine Mammary Tumor Cell Lines Associated with Aberrant LEF1 Expression
Source: PLoS One. 2014 Jun 2;9(6):e98698. doi: 10.1371/journal.pone.0098698 (PMC4041801; doi:10.1371/journal.pone.0098698)

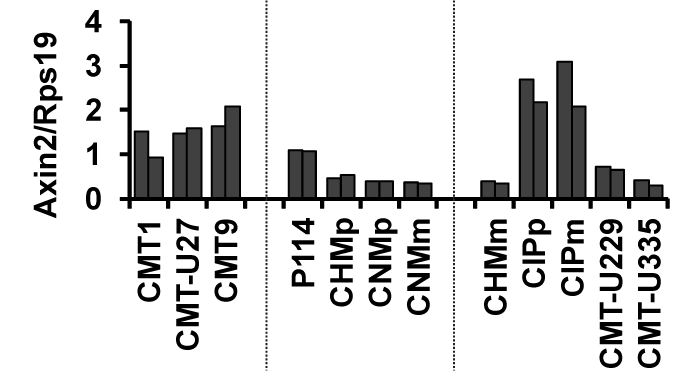

Supplement: Figure S1 — Axin2 mRNA expression. Rps19 normalized Axin2 mRNA expression in two different passages of canine mammary cell lines. Cell lines were divided in three groups (from left to right): cell lines with high, moderate or absent canonical Wnt activity. (TIF) [file pone.0098698.s001.tif]

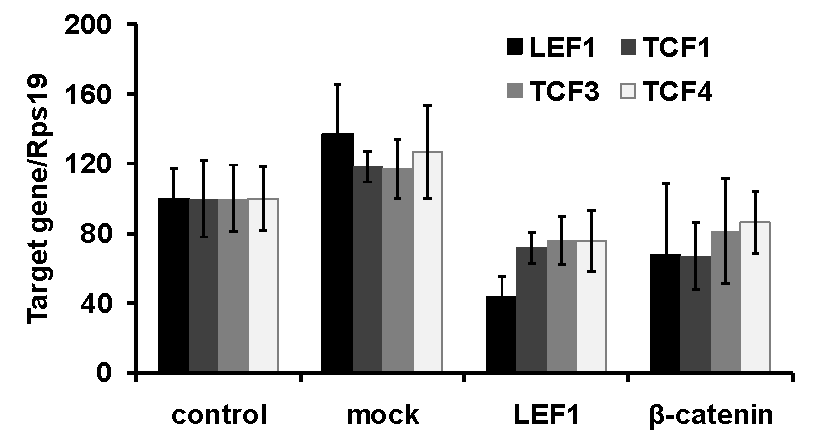

Supplement: Figure S2 — Expression of TCF-family members upon LEF1 knock-down. Relative Rps19 normalized mRNA expression of LEF1, TCF1, TCF3 and TCF4 24 h after LEF1 knock-down in CMT-U27 cells. Average expression of control conditions for each target gene is set to 100. (TIF) [file pone.0098698.s002.tif]
