# Supplementary material for: Ligand-Independent Canonical Wnt Activity in Canine Mammary Tumor Cell Lines Associated with Aberrant LEF1 Expression
Source: PLoS One. 2014 Jun 2;9(6):e98698. doi: 10.1371/journal.pone.0098698 (PMC4041801; doi:10.1371/journal.pone.0098698)
Supplement: Table S1 — Sequencing results of target gene coding regions in canine mammary tumor cell lines. (DOCX) [file pone.0098698.s003.docx]

| **Transcript** | **β-catenin** | **APC*** | **GSK3β** | **Axin1** | **CK1α** | **CDH1** | **LEF1** |
| --- | --- | --- | --- | --- | --- | --- | --- |
| ref seq | NM_001137652.1 | XM_536285 | XM_851518.2 | XM_847228 | XM_536470 | XM_536807 | XM_858241 |
| **CMT1** | wt | wt | snp 1807 GGG->GGA | 50% snp 1753 GCA->GCG  snp 2344 GCC->GCT  snp 2550+2551 TCA->TTC (S->F)  snp 2658…2666 ACA GGT GGA TGA-> AAG GTG GAC TGA (T G G - -> L V D -) | wt | snp1743 ACG>ACA | Missing exon 6 |
| **CMT-U27** | wt | wt | snp 1807 GGG->GGA | 50% snp 1753 GCA->GCG  snp 2344 GCC->GCT  snp 2550+2551 TCA->TTC (S->F)  snp 2658…2666 ACA GGT GGA TGA-> AAG GTG GAC TGA (T G G - -> L V D -) | wt | snp1743 ACG>ACA | Missing exon 6 |
| **CMT9** | wt | wt | snp 1807 GGG->GGA | 50% snp 1753 GCA->GCG  snp 2344 GCC->GCT  snp 2550+2551 TCA->TTC (S->F)  snp 2658…2666 ACA GGT GGA TGA-> AAG GTG GAC TGA (T G G - -> L V D -) | wt | snp1743 ACG>ACA | Missing exon 6 |
| **P114** | wt |  |  | snp 2550+2551 TCA->TTC (S->F)  snp 2658…2666 ACA GGT GGA TGA-> AAG GTG GAC TGA (T G G - -> L V D -) |  | snp1743 ACG>ACA |  |
| **CHMp** | 50% snp 1770 GCC->GCT  50% snp 2134 CTT->ATT (L->I) |  |  |  |  | snp1743 ACG>ACA |  |
| **CHMm** | 50% snp 1770 GCC->GCT  50% snp 2134 CTT->ATT (L->I) |  |  |  |  | snp1743 ACG>ACA |  |
| **CIPp** | wt |  |  | snp 2550+2551 TCA->TTC (S->F)  snp 2658…2666 ACA GGT GGA TGA-> AAG GTG GAC TGA (T G G - -> L V D -) | wt |  | wt |
| **CIPm** |  |  |  | snp 2658…2666 ACA GGT GGA TGA-> AAG GTG GAC TGA (T G G - -> L V D -) |  | snp1743 ACG>ACR |  |
| **CNMp** | 50% snp 1770 GCC->GCT  50% snp 2134 CTT->ATT (L->I) |  |  | snp 2550+2551 TCA->TTC (S->F)  snp 2658…2666 ACA GGT GGA TGA-> AAG GTG GAC TGA (T G G - -> L V D -) |  |  |  |
| **CNMm** | 66%snp 1770 GCC->GCT |  |  |  |  |  |  |

*mutation cluster region and the downstream sequence

wt = wild-type, SNP = single nucleotide polymorphism, underlined = changes affecting protein sequence
